# Reply to: Comment on “Late cachexia or treatment-related muscle wasting in long-term HNSCC survivors?”

**DOI:** 10.1016/j.clinsp.2026.101137

**Published:** 2026-09-10

**Authors:** Willian das Neves

**Affiliations:** Instituto do Câncer do Estado de São Paulo (ICESP), Hospital das Clínicas, Faculdade de Medicina, Universidade de São Paulo (HCFMUSP), São Paulo, SP, Brazil

Dear Editor,

We thank Topkan and colleagues for carefully reading our article and for generously recognizing the relevance of studying muscle abnormalities in long-term, disease-free Head and Neck Squamous Cell Carcinoma (HNSCC) survivors.^1^^,^^2^ Constructive exchanges like this are exactly what this underexplored field requires, and we welcome the opportunity to clarify the points raised. In our view, several concerns reflect deliberate design features of a survivorship study, and, most importantly, the conceptual concern about the label “cancer cachexia” speaks less to our study than to the limitations of the diagnostic frameworks currently available to the field.

Regarding sampling and selection, we respectfully submit that the “survivor-enriched” composition of our cohort is an inherent feature of the population under investigation rather than a source of bias within the defined target population. It does, however, limit external validity to comparable long-term survivor populations. Our research question addressed the prevalence of cachexia among disease-free patients at least two years after curative chemoradiotherapy; by definition, this population includes only long-term survivors, as in studies of any late toxicity (e.g., osteoradionecrosis, hypothyroidism, or late dysphagia), where conditioning on survival is inherent to the design. Within the eligible population, we recruited consecutively to mitigate selection bias. We made no claim that our estimates extrapolate to the broader HNSCC population at diagnosis; instead, our discussion explicitly restricted generalizability to comparable survivor populations treated at tertiary oncology centers.^2^

We agree that the small absolute number of cachectic patients limits statistical power and widens confidence intervals, particularly for analyses stratified by the Evans criteria (n = 10). As stated in the manuscript, no formal sample size calculation was performed; the study was descriptive and hypothesis-generating, and null findings, including the absence of an association between cachexia and dysphagia or biochemical markers, should be interpreted as absence of evidence rather than evidence of absence. To our knowledge, however, these are the first prevalence data obtained more than two years after curative chemoradiotherapy, and on this basis we called for larger cohorts in our conclusion.^2^

We welcome our colleagues' suggestion to conduct a formal concordance analysis between the Fearon^3^ and Evans^4^ criteria, and we performed it post hoc. Among the 111 patients evaluable under both definitions, the two criteria identified largely different individuals: of the 26 classified as cachectic by either definition, only 7 met both (Cohen's κ = 0.34, 95% CI 0.12–0.56), and the two definitions yielded significantly different prevalence estimates (McNemar exact test, p = 0.004).

These findings do not, however, indicate a measurement artifact of our cohort: discordance between cachexia definitions is a reproducible, field-wide phenomenon. When three published cachexia classification systems were applied to 1384 patients with incurable cancer, only 3.1% were classified as cachectic by all three.^3^ Even within the Fearon framework, merely substituting the instrument used to estimate muscle mass shifted cachexia prevalence from 21.8% to 32.2% in a multicenter cohort of 5110 patients.^4^ The Fearon and Evans definitions were based on partially distinct constructs (weight loss and body composition in one case, and a multidimensional phenotype including strength, fatigue, anorexia, and biochemistry in the other), so their divergence is not unexpected. The consensus authors recommended formal validation of these frameworks, but it remains incomplete.^5^

We deliberately applied both definitions to show how strongly prevalence estimates among survivors depend on the chosen instrument. The two most widely used definitions identify partially overlapping but non-identical patient populations, and we believe this finding argues for harmonized, survivor-specific criteria. The consensus anticipated this, noting that “there are special populations for which the definition and diagnostic criteria might need specific modification”, and that this “would have to be part of the general validation process”.^5^ Long-term disease-free survivors are precisely such a population, a conclusion on which our colleagues, the consensus authors, and we appear to converge.

The central conceptual concern ‒ that normal hemoglobin, albumin, and C-Reactive Protein (CRP) levels make the label “cancer cachexia” problematic, favoring “treatment-related muscle wasting” ‒ warrants a direct response. We classified our patients using the two most commonly adopted published criteria. The international consensus definition requires no inflammatory biomarker for diagnosis, and its authors are explicit on the point: “The most widely accepted index of systemic inflammation is serum C-Reactive Protein (CRP). However, cachexia can exist without overt systemic inflammation”.^5^ Importantly, the same consensus was explicit about the provisional status of its thresholds: because no trials with demonstrated efficacy in cancer cachexia were available to inform them, “the criteria suggested in the present consensus remain arbitrary”.^5^ The indirect indices of catabolic drive that the consensus recommends when CRP is unrevealing ‒ responsiveness to chemotherapy and rate of disease progression ‒ are, by construction, inapplicable to disease-free survivors, illustrating how far these criteria are from the population we studied.

In the Evans definition, abnormal biochemistry is one of five minor criteria, any three of which suffice.^6^ Under the currently available operational definitions, these patients meet criteria for cachexia; if meeting published criteria is deemed insufficient for the diagnosis, the limitation lies with the criteria rather than with their application. The Evans panel acknowledged as much, stating that “this definition has not been tested in epidemiological or intervention studies” and listing among its open questions whether “cytokines or inflammation [are] central to the pathophysiology of cachexia”.^6^ The absence of elevated CRP therefore does not exclude the underlying metabolic and muscle-wasting phenotype.^5^^,^^7^

On the underlying biology, we substantially agree with our colleagues: a treatment-related component is plausible and was explicitly discussed in our article, including cisplatin-induced mitochondrial myotoxicity and imaging evidence of radiotherapy-associated muscle depletion.^2^ We deliberately used the term “late cachexia” to provoke the very discussion they now advance. Patients who meet current criteria years after cure challenge the assumption that cachexia requires ongoing tumor-host interaction, an assumption that remains unsettled, since “Does treatment of the underlying illness completely resolve cachexia?” was listed as an open research question by the Evans panel itself.^6^ Cachexia diagnosed by established criteria has been documented in disease-free HNSCC patients from the immediate post-treatment period onward,^8^ and sarcopenia in this population predicts both survival and late toxicity.^9^ Whether the phenotype is best conceptualized as late cachexia, treatment-related wasting, accelerated sarcopenia, or a combination cannot presently be adjudicated: the contemporary frameworks that distinguish these entities remain conceptual and offer no validated, operational criteria applicable to disease-free survivors. Our data expose exactly this gap.

Finally, we fully agree that the cross-sectional design precludes causal inference regarding the origin and trajectory of muscle depletion. Accordingly, we made no causal claims, and we reported the study in accordance with STROBE recommendations for cross-sectional studies. We acknowledged the lack of computed tomography-based muscle quantification as our first limitation and share the view that longitudinal, population-representative cohorts with serial imaging are the necessary next step. Our prevalence estimates, in effect, justify establishing such cohorts.^2^

In summary, we believe the points of genuine divergence are few. Our colleagues and we agree that persistent muscle loss and dysfunction in disease-free HNSCC survivors are real, clinically relevant, and insufficiently characterized; that current cachexia definitions perform poorly in this population; and that longitudinal studies with standardized muscle imaging and phenotypic distinction among cachexia, sarcopenia, and treatment-related wasting are required. We thank Topkan and colleagues for enriching this discussion and Clinics for hosting it.

## Ethical approval

The Human Research Ethics Committee of the University of São Paulo approved the original study (CEP-FMUSP; protocol 1.339.832). No new data were collected for this correspondence.

## Declaration of generative AI and AI-assisted technologies in the manuscript preparation process

During the preparation of this work, the author used Claude (Anthropic) to support literature retrieval, drafting, and language editing. The argumentative framework and scientific content are the author's; the author verified all sources and data and takes full responsibility for the content of the published article.

## Funding

No specific funding was received for this correspondence.

## Declaration of competing interest

The author declares no conflicts of interest.

## Data Availability

The data supporting this correspondence are those of the original study and are available from the corresponding author upon reasonable request.
